# Effect of TADF Assistance on Performance Enhancement in Solution Processed Green Phosphorescent OLEDs

**DOI:** 10.3390/polym13071148

**Published:** 2021-04-02

**Authors:** Ewelina Witkowska, Gabriela Wiosna-Salyga, Ireneusz Glowacki, Tung-Huei Ke, Pawel Malinowski, Paul Heremans

**Affiliations:** 1 Department of Molecular Physics, Lodz University of Technology, Zeromskiego 116, 90-924 Lodz, Poland; ewelina.witkowska@p.lodz.pl (E.W.); ireneusz.glowacki@p.lodz.pl (I.G.); 2 IMEC, Kapeldreef 75, B-3001 Leuven, Belgium; pawel.malinowski@imec.be (P.M.); paul.heremans@imec.be (P.H.)

**Keywords:** organic light emitting diode, TADF assistant dopant, exciplex, energy transfer, charge carrier trapping, thermolumienscence

## Abstract

Many methods have been proposed to increase the efficiency of organic electroluminescent materials applied as an emissive layer in organic light emitting diodes (OLEDs). Herein, we demonstrate enhancement of electroluminescence efficiency and operational stability solution processed OLEDs by employing thermally activated delayed fluorescence (TADF) molecules as assistant dopants in host-guest systems. The TADF assistant dopant (SpiroAC–TRZ) is used to facilitate efficient energy transfer from host material poly(*N*–vinylcarbazole) (PVK) to a phosphorescent Ir(III) emitter. We present the analysis of energy transfer and charge trapping—two main processes playing a crucial role in light generation in host–guest structure OLEDs. The investigation of photo-, electro- and thermoluminescence for the double-dopant layer revealed that assistant dopant does not only harvest and transfer the electrically generated excitons to phosphorescent emitter molecules but also creates exciplexes. The triplet states of formed PVK:SpiroAC–TRZ exciplexes are involved in the transport process of charge carriers and promote long–range exciton energy transfer to the emitter, improving the efficiency of electroluminescence in a single emissive layer OLED, resulting in devices with luminance exceeding 18 000 cd/m^2^ with a luminous efficiency of 23 cd/A and external quantum efficiency (EQE) of 7.4%.

## 1. Introduction

Thermally activated delayed fluorescence (TADF) type materials have been lately considered as new promising emitters for organic light emitting diodes (OLEDs) [1,2,3]. This type of fluorescent material enables efficient triplet harvesting, that in the past was primarily achieved in OLEDs with phosphorescent emitters. In that case, it is possibly due to a small difference between the energy of the lowest singlet (S_1_) and triplet (T_1_) excited states that enables reverse intersystem crossing (RISC) from the triplet to the singlet state. Small triplet–singlet energy splitting (Δ*E*_ST_) might be overcome by excitons with the aid of minimal thermal energy. Therefore, this process is efficient even at room temperature. Consequently, OLED performance based on TADF materials might be comparable to those based on phosphorescent emitters [4].

The fundamental feature of TADF molecules, i.e., small Δ*E*_ST_, could be obtained by separation of frontier molecular orbitals through the application of the donor–acceptor (D–A) molecular structure [5]. Therefore, TADF materials are mostly obtained by linking A and D units via an aromatic bridge. In such a case, the highest occupied molecular orbital (HOMO) is mostly located on a donor part, whereas the lowest occupied molecular orbital (LUMO) is placed on an acceptor [6]. Separation of D and A units supports electron transfer in the excited state between the units, consequently increasing charge transfer (CT) character.

The majority of TADF based OLEDs are prepared by vacuum deposition. Solution processing is generally unsuitable for TADF materials since they usually suffer from low solubility and poor film formation ability. To solve this problem, different molecular architectures of TADF, like dendrimers and polymers, were proposed [7]. However, it is still quite challenging to develop materials that can fully meet key requirements for solution processable OLEDs. The small TADF molecules normally do not create an amorphous layer, whereas the incorporation of TADF in a solution-processable matrix usually affects the dynamics of the excited states that leads to large changes in the emission spectrum and quantum yield (QY). Furthermore, phase separation is another problem due to differences in the molecular structures of the materials. However, a few reports about solution processed OLEDs that were based on host–TADF guest have appeared lately [8,9,10].

**Figure 1:**
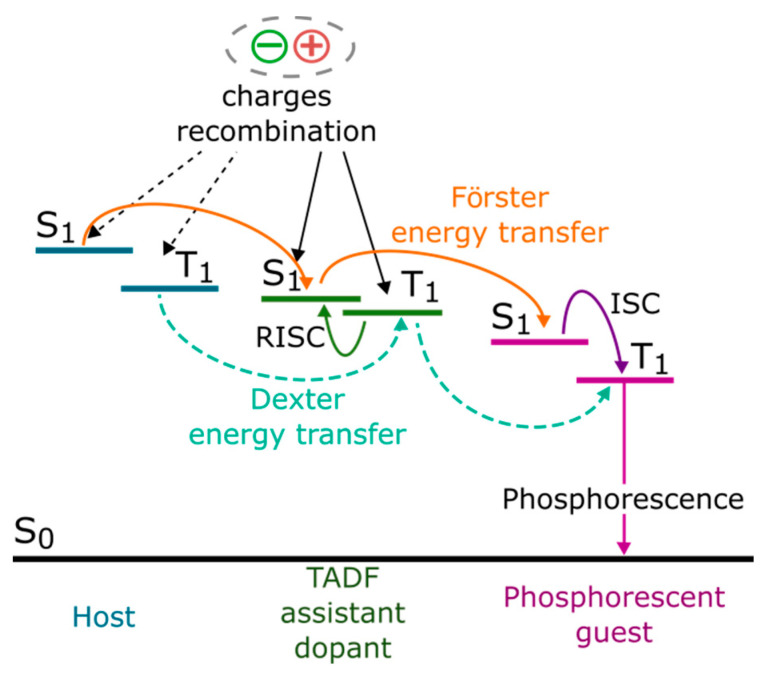
The energy transfer diagram of light emitting system with thermally activated delayed fluorescence (TADF) assistant dopant and phosphorescent emitter.

TADF materials can be used in OLEDs not only directly as highly efficient emitters. The fact that they have a small energy gap between the singlet and the triplet energy states (Δ*E*_ST_) makes them good candidates for the host in which triplets are effectively harvested. Several reports about TADF material application as a host for fluorescent emitter have been published [11]. Furthermore, TADF hosts have been compiled not only with fluorescent, but also phosphorescent and even TADF emitters [11]. In such systems, triplet excitons created on the TADF host are converted to singlet states and then the exciton energy might be transferred via long-range Förster energy transfer to the guest. This allows for small doping concentration and a reduction of emitter concentration quenching effect. Furthermore, triplet excited states of the host are effectively employed and triplet–triplet annihilation is reduced [12]. However, in such an emissive layer, it is quite difficult to obtain balanced charge transport properties. Therefore, this approach was modified and the standard materials with the TADF working as an assistant dopant for fluorescent OLEDs were proposed [13]. TADF assistance could be also applied in the case of a phosphorescent target emitter [14,15]. Possible energy transfer pathways in such systems are schematically depicted in Figure 1. When the TADF assistant dopant is introduced to the host–guest system, two mechanisms can be considered. One of them is devoted to exciton formation directly on TADF molecules. On the other hand, charge carrier recombination may occur on host molecules, and consequently, excitons created on the matrix are subsequently transferred to the TADF molecules through the S_1_ (host) → S_1_ (assistant) or T_1_ (host) → T_1_ (assistant) route (via Förster as well as Dexter mechanisms) [14]. In these models, triplet excitons generated on the assistant dopant are converted to TADF singlet excitons by reverse intersystem crossing (RISC), and following Förster energy transfer, favor the creation of excited S_1_ states of the guest. Finally, efficient emission from the guest emitter molecules can occur. For both types of emitters (fluorescent and phosphorescent), Dexter energy transfer can also operate; however, in the case of the former emitter, Dexter energy transfer T_1_ (assistant) → T_1_ (emitter) leads to losses due to non-emissive triplet states. On the contrary, the application of phosphorescent emitter allows effective utilization of both pathways.

Although this approach can increase the efficiency of light generation, application of such a complicated system in solution processed OLEDs remains challenging, mainly because of the relatively high TADF concentration (15–50%) that is required in the system [13].

This work demonstrates a strategy to optimize the efficiency of electroluminescence of single emitting layer OLEDs through introducing TADF molecules as an assistant dopant in host–guest systems. The photo-, electro- and thermoluminescence studies were carried out to determine the role of the TADF assistant dopant in the energy transfer and charge trapping processes and its influence on the performance of devices. The subject of this study is the modified host–guest emissive layer composed of host material assisted with TADF dopant and phosphorescent guest emitter. One of the recently synthetized iridium (III) complexes: [bis(benzo[*h*]quinolinato-*N*,*C*^10^′){4-((1-naphthyl)imino)-pent-2-en-2-olato-*N,O*} iridium(III)] (Ir) [16] has been studied as a guest emitter. This Ir complex has been proven as an efficient green emitter in host–guest type OLEDs that were based on the matrix mixture of poly(*N*–vinylcarbazole) (PVK) with oxadiazole derivative (PBD) [16]. In order to apply a challenging two-doped host–guest system (matrix–assistant dopant–emitter), firstly, a single-component matrix (PVK) was studied. Additional attempts have been made to use as a host the PVK:PBD mixture mentioned above. To meet the basic requirements for efficient energy transfer from the TADF to the target green emitter, 10-(4-(4,6-diphenyl-1,3,5-triazin-2-yl)phenyl)-10H-spiro[acridine-9,9’-fluorene] (SpiroAC–TRZ) with high triplet energy level and blue emission [17] has been proposed as an assistant dopant.

## 2. Materials and Methods

### 2.1. Materials

**Figure 2:**
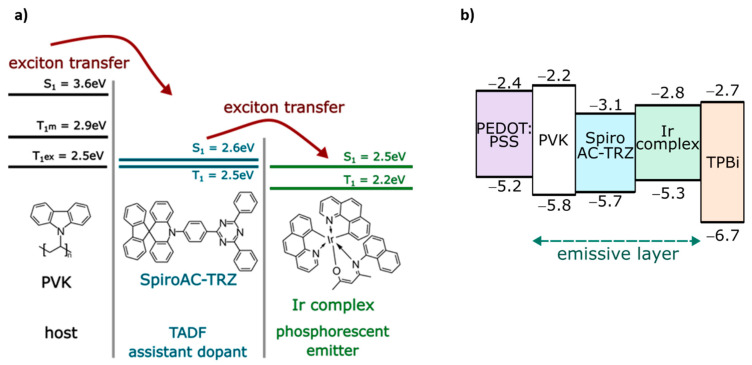
Scheme representing (**a**) singlet and triplet energies of emissive layer components (Tm indicates the triplet of monomer and Tex- triplet of excimer) and (**b**) relations between the highest occupied molecular orbital (HOMO) and the lowest occupied molecular orbital (LUMO) levels of applied poly(*N*–vinylcarbazole) (PVK) matrix [19] for SpiroAC–TRZ [17] assistant dopant and phosphorescent emitter –Ir complex [16] in organic light emitting diode (OLED) configuration, where poly(3,4–ethylenedioxythiophene) and poly(styrenesulfonate) mixture (PEDOT:PSS) [20] works as hole injection layer (HIL) and 1,3,5-tris(N-phen-ylbenzimidazol-2-yl)-benzene (TPBi) [21] as electron transport layer (ETL).

As a host, poly(*N*-vinylcarbazole) (PVK) (M_w_ = 1.1 × 10^6^ g/mole) was purchased from Sigma-Aldrich (St Louis, MO, USA) and used as received. The TADF emitter, i.e., 10-(4-(4,6-diphenyl-1,3,5-triazin-2-yl)phenyl)-10H-spiro[acridine-9,9’-fluorene] SpiroAC–TRZ) was purchased from Lumtec (New Taipei City, Taiwan). In SpiroAC–TRZ, intramolecular charge transfer transition occurs from the acridine donor unit to the triazine moiety (see Figure 2). HOMO and LUMO levels of SpiroAC–TRZ are −5.7 eV and −3.1 eV, respectively, whereas singlet–triplet splitting was estimated to be 0.07 eV [18].

The iridium (III) complex ([bis(benzo[*h*]quinolinato-*N*,*C*^10^′){4-((1-naphtyl)imino)-pent-2-en-2-olato-*N,O*}iridium(III)]) synthesis route and photophysical properties were published in our previous work [16].

### 2.2. Spectroscopic Measurements

Photophysical studies were performed in a solution as well as for emissive layers. All compounds were diluted in chlorobenzene. Thin films of PVK + TADF + Ir blends were prepared on quartz substrates by means of spin-coating. Absorption spectra were detected by a Cary 5000 (Varian Inc.) spectrometer (Palo Alto, CA, USA). The emission spectra were recorded on an Edinburgh Instruments FLS980 spectrofluorometer (Livingston, UK) equipped with an integrating sphere used to determine the photoluminescence quantum yield.

Samples of thick layers (few µm), required for spectrally resolved thermoluminescence (SRTL) experiments, were prepared by drop-casting and spin-coating from chlorobenzene solutions onto aluminum substrates. The samples for SRTL studies were placed in the vacuum chamber on a thermostated holder and covered by a sapphire plate. After sample photoexcitation at 15 K by pulsed nitrogen laser (λ = 337 nm) (PTI, model GL–3300T), the thermoluminescence (TL) measurements were carried out in the temperature range of 20–300 K with a heating rate of 7 K/min. Sample thermoluminescence was recorded by a detection system contained an optical collector, an optical-fiber, a Micro HR Imaging Spectrograph and a CCD 3500 camera (Horriba Jobin–Yvon).

### 2.3. Fabrication and Characterisation of OLEDs

The OLEDs were fabricated on glass ITO substrates by means of the spin-coating technique followed by vacuum deposition. Firstly, the hole injection layer of poly(3,4–ethylenedioxythiophene) and poly(styrenesulfonate) mixture (PEDOT:PSS) was spin coated on indium tin oxide (ITO) anode in ambient conditions. Secondly, the light emitting layer (LEL) consisted of PVK + *x* wt% TADF with or without Ir complex was spin–coated from a chlorobenzene solution in a glovebox. The assistant dopant concentration influence on the device parameters were checked in the range of 15–30 wt%. As the last step, the following layers were patterned through a shadow mask by the physical vacuum deposition technique. 1,3,5-tris(*N*-phen-ylbenzimidazol-2-yl)-benzene (TPBi) was applied as electron transport layer, whereas (8-quinolinolato)lithium (Liq) as electron injecting material. The device was covered by silver (Ag) cathode. Generally, the complete device stack can be written as: ITO/PEDOT:PSS (20 nm)/LEL (65 nm)/TPBi (20 nm)/Liq (2 nm)/Ag (100 nm). Additionally, for the best composition of the emissive layer, OLED configuration was checked with a 4,7-diphenyl-1,10-phenanthroline (Bphen) electron transporting layer. The device *J*–*V*–*L* characteristics were determined with the use of a Keithley 237 source measurement unit connected with a Minolta CS–2000a camera.

## 3. Results

Singlet and triplet energies of the components of the studied emissive layer are depicted in Figure 2. The energy cascade shown should ensure efficient energy transfer into the Ir complex. Additionally, the relative position of HOMO and LUMO levels of emissive materials and materials used for the hole injection layer (HIL) and an electron transport layer (ETL) are presented. Due to the hole transporting properties of the applied PVK matrix, the ETL was introduced to improve a charge carriers balance in the emissive system. According to the energetic alignment, ETL (1,3,5-tris(*N*-phen-ylbenzimidazol-2-yl)-benzene (TPBi)) can simultaneously act as a hole blocking layer, which may result in an increase of emission efficiency.

### 3.1. Photophysical Properties and OLEDs Parameters

**Figure 3:**
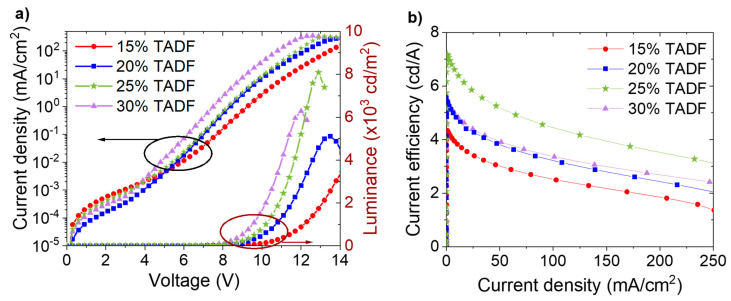
Working parameters of OLEDs based on PVK doped with different SpiroAC–TRZ (TADF) concentration: (**a**) *J*–*V*–*L* characteristics: current density–voltage characteristics (left) and luminance–voltage characteristics (right); (**b**) Current efficiency vs current density. OLED configuration: ITO/PEDOT:PSS (20 nm)/light emitting layer (LEL) (70 nm)/TPBi (20 nm)/Liq (2 nm)/Ag (100 nm).

In order to design the efficient multicomponent active layer, SpiroAC–TRZ content was adjusted as the first step. Therefore, photoluminescence (PL) and electroluminescence (EL) studies were performed on emissive layers composed only of matrix and TADF material, without a phosphorescent emitter. A change in the SpiroAC–TRZ content in PVK, in the range of 15–30 wt%, has a negligible effect on PL spectra (Figure S1a). Only the emission band associated with SpiroAC–TRZ molecules is visible, whose maximum is slightly shifted from 486 to 494 nm with an increasing amount of TADF molecules (Table 1). The PL quantum efficiency (QY) of these emissive layers practically does not depend on SpiroAC–TRZ content and is equal to 46% in the range of 15–25 wt% of the TADF material. Only for the highest concentration (30 wt%) does it drop to 42% (Table 1), which can be associated with the intermolecular quenching effect. Interestingly, the assistant dopant concentration noticeably influenced EL spectra shape (Figure S1b) as well as the value of external efficiency. The emission band is widened in the long-wave range with increased content of SpiroAC–TRZ, that affects slightly the color of emitted light (the insert in Figure S1b). This may indicate that for layers with higher SpiroAC–TRZ content, some additional emissive states may participate in the EL phenomenon (the nature of which is discussed later in the article). Current density–luminance–voltage (*J*–*V*–*L*) characteristics of OLEDs with emitting layers, based on PVK and different SpiroAC–TRZ content, are presented in Figure 3a. One can see typical OLED characteristics in the range up to supply voltage of 14 V. The turn-on voltage (the voltage at which the luminance reaches 1 cd/m^2^) decreases with an increase of SpiroAC–TRZ in PVK, from 6.9 V for 15 wt% to 5.4 V for 30 wt% (Figure S2). Since the thickness of the light-emitting layer (LEL) has been kept constant (about 70 nm), this effect can be explained by easier injection of charge carriers directly into the TADF molecules and consequently excitons generation promptly on them. The highest luminance value (over 8 000 cd/m^2^) (Figure 3a), and the highest current efficiency, above 7 cd/A (Figure 3b), were obtained for devices based on PVK with 25 wt% of SpiroAC–TRZ. It should be noted that a further increase of TADF content up to 30 wt% results in the lowering of luminance and device performance. Probably, concentration quenching starts to play a role. A similar effect was previously observed by Lin and et al. [17].

**Figure 4:**
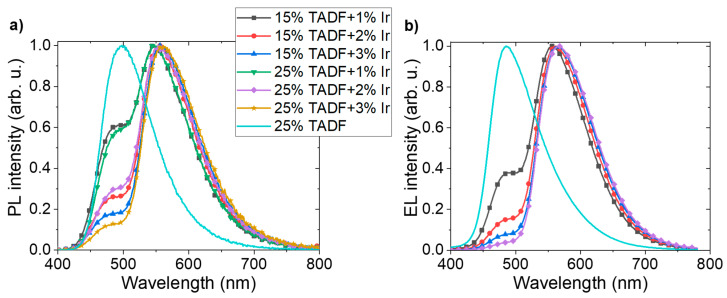
(**a**) PL spectra of PVK layers with different SpiroAC–TRZ (15 or 25 wt%) and Ir complex (Ir) (1–3 wt%) concentrations; (**b**) EL spectra of OLEDs based on the investigated systems. OLED configuration: ITO/PEDOT:PSS (20 nm)/LEL (70 nm)/TPBi (20 nm)/Liq (2 nm)/Ag (100 nm).

The most efficient emissive system in OLEDs (PVK with 25 wt% of SpiroAC–TRZ) and the one with the lowest TADF content (15 wt%) were then doped with the final phosphorescent emitter. PL spectra of layers consisting of PVK:SpiroAC–TRZ with 1–3 wt% of Ir are presented in Figure 4a. It is visible that the exciton energy of the assistant dopant is not fully transferred, even in systems with the largest Ir complex concentration. It is shown in Figure 4a that the PL intensity of the TADF emission (peak at 500 nm) in the PL spectra of layers with 15 and 25 wt% of SpiroAC–TRZ and the same concentration of the Ir complex are similar. The lowest of TADF emission was observed for PVK + 25 wt% SpiroAC–TRZ + 3 wt% Ir system. Therefore, it can be assumed that the energy transfer to the phosphorescent dopant in studied emissive layers does not depend on the TADF concentration and is more efficient when the content of Ir complex in the layer is higher. The situation is a little bit different in the case of electric excitation. It is worth underlining that in EL spectra, the contribution of TADF emission is lower than in PL spectra (compare Figure 4 a,b) and the amount of TADF assistant molecules seems to be more important for emissive efficiency of the layer. Although the highest QY (27%) has been obtained for the layer of PVK + 15 wt% SpiroAC–TRZ + 2 wt% Ir, the best efficient devices were obtained with the PVK + 25 wt% SpiroAC–TRZ + 2 wt% Ir emissive system (Table 1). Despite the lowest contribution of TADF emission having been observed in systems with 3 wt% of Ir complex (Figure 4a), the QY values determined for these layers start to decrease (to 21 and 19%), probably due to the effect of concentration quenching of the emissive states, which is typically observed for iridium (III) complexes [22,23].

**Figure 5:**
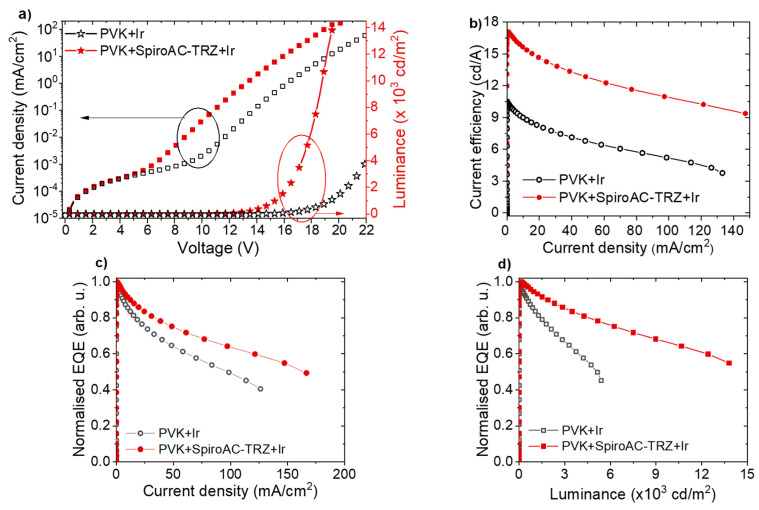
Working parameters of OLEDs with the following structure: ITO/PEDOT:PSS (20 nm)/LEL (70 nm)/TPBi (40 nm)/Liq (2 nm)/Ag (100 nm), where LEL is based on PVK+2wt% Ir (black) or PVK+25 wt% SpiroAC–TRZ+2 wt% Ir (red); (**a**) *J*–*V*–*L* characteristics, (**b**) current efficiency vs current density, (**c**) EQE (external quantum efficiency) vs current density, (**d**) EQE vs luminance.

The impact of SpiroAC–TRZ assistance on OLED parameters is depicted in Figure 5. For direct comparison, results for OLEDs with and without assistant dopant are shown in the same plots. It is visible that the addition of TADF material led to luminance and efficiency increase with simultaneous lowering of turn-on voltage (Figure 5a,b). Additionally, implementation of TADF material significantly reduced efficiency roll-off (Figure 5c,d), probably by preventing the triplet–triplet annihilation process by subsequent exciton transfer to the iridium complex molecules [24]. It should be clarified that optimization of OLED construction has shown that devices with thicker ETL had better reproducibility and stability (Figure S3); therefore, in the further investigations, ETL thickness was increased from 20 to 40 nm.

Finally, the emissive layer composed of PVK + 25 wt% SpiroAC–TRZ + 2 wt% **Ir** was assumed to be the best and further investigations were performed on it.

**Figure 6:**
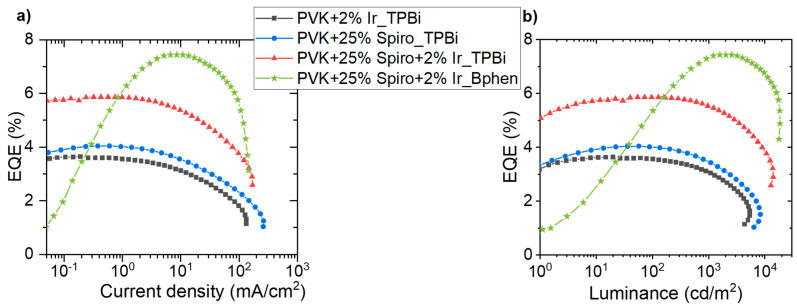
Working parameters of OLEDs with different emissive layers: PVK + 2 wt% Ir, PVK + 25 wt% SpiroAC–TRZ and PVK + 25 wt% SpiroAC–TRZ + 2 wt% Ir. Device structure: ITO/PEDOT:PSS(20 nm)/LEL(65 nm)/TPBi or Bphen(40 nm)/Liq(2 nm)/Ag(100 nm). (**a**) EQE vs current density; (**b**) EQE vs luminance.

Results presented so far were obtained for devices with 1,3,5-tris(N-phen-ylbenzimidazol-2-yl)-benzene (TPBi) applied as ETL. Additionally, 4,7-diphenyl-1,10-phenanthroline (Bphen) was checked as an alternative to TPBi. In both cases, thicker ETL (40 nm) and 70−65 nm emission layer thickness guaranteed better device stability. To evaluate the performance of Bphen ETL, OLEDs with optimized device structure of ITO/ PEDOT:PSS (20 nm)/ LEL (65 nm)/ Bphen (40 nm)/ Liq (2 nm)/ Ag (100 nm) were fabricated. Bphen exhibits LUMO = −3.0 eV, that is, lower in comparison to the LUMO of TPBi (−2.8 eV) [21]. Moreover, its electron mobility is around one order of magnitude higher (μ*_e_*_(Bphen)_ = 3.2 × 10^−4^ cm^2^ V^−1^ s^−1^, μ*_e_*_(TPBi)_ = 3.3–8 × 10^−5^ cm^2^ V^−1^ s^−1^) [25,26]. Comparing results with different ETLs (Figure S4), one can see that Bphen is significantly better for the emissive system with the TADF assistant dopant. The HOMO level of Bphen (−6.5 eV) [21] is 0.3 eV lower than the HOMO of TPBi (−6.2 eV), which promotes hole carriers accumulation at the interface between LEL and ETL, taking into account that the matrix (PVK) is a hole transporting material. In addition, the lower level of Bphen LUMO (−3.0 eV) supports the easier injection of electrons from the cathode into the ETL. Considering the above and the fact that the electron mobility of Bphen is higher, an increase in current density and lowering of turn-on voltage could be expected. Additionally, the better charge carries balance in device increases luminance and current efficiency. As it is seen in Figure 6, all of these effects contributed to device performance improvement. Interestingly, the same shapes of OLEDs characteristics—external quantum efficiency (EQE) vs current density and EQE vs luminance—were observed independently of applied LEL (PVK + 2 wt% Ir, PVK + 25 wt% SpiroAC–TRZ, PVK + 25 wt% SpiroAC–TRZ + 2 wt% Ir) for devices with TPBi ETL. Nonetheless, completely different dependence was recorded for Bphen implementation. At the start of device operation (*J* < 5 mA/cm^2^ and *Lv* < 100 cd/m^2^), rapid growth of EQE is observed, whereas in the case of TPBi application, this value is relatively constant in the range of low current density. However, for OLEDs with Bphen layer, EQE value does not significantly change in the current density range of 10–70 mA/cm^2^ and luminance range of 1000–1500 cd/m^2^ (Figure 6), which is more advantageous in terms of application.

**Figure 7:**
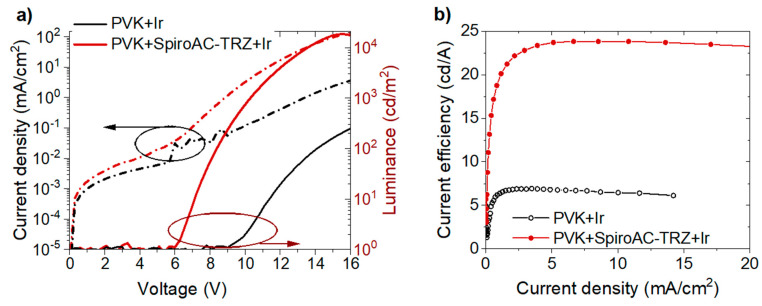
Working parameters of OLEDs with emissive layers: PVK + 2 wt% Ir and PVK + 25 wt% SpiroAC–TRZ + 2 wt% **Ir**. Device structure: ITO/PEDOT:PSS (20 nm)/LEL (65 nm)/Bphen (40 nm)/ Liq (2 nm)/Ag (100 nm). (**a**) Current density–voltage–luminance (*J*–*V*–*L*) characteristics; Current density–voltage characteristics (left) and luminance–voltage characteristics (right); (**b**) Current efficiency vs current density.

In order to sum up TADF assistance, a comparison of OLEDs parameters with Bphen ETL and the emissive layers composed of PVK+2 wt% Ir or PVK + 25 wt% SpiroAC–TRZ + 2 wt% Ir is depicted in Figure 7. As one can see, SpiroAC–TRZ addition resulted in lowering of turn-on voltage, rising brightness, stabilizing flowing current and four-times increase of current efficiency. The influence of the TADF assistant is similar independently of the used ETL material although the more considerable improvement was obtained in OLEDs with a Bphen layer. It should be stressed that different values of turn-on voltage, observed in *L*–*V* characteristics (*c.f.* Figure 5a and Figure 7a), do not arise from the device thickness which was kept similar for the tested diodes. Furthermore, in all cases, EL completely occurs from the Ir complex, that confirms appropriate materials adjustment (Figure S5c and [16]). The parameters of all devices described in this section are compared in Table S1.

### 3.2. Spectrally Resolved Thermoluminescence Studies

Charge trapping and energy transfer are the two crucial processes in the light generation in OLEDs based on a host–guest emissive system. It is interesting to evaluate the influence of a large amount of TADF molecules on charge carriers trapping processes in the PVK matrix. Such processes can be investigated employing the thermoluminescence (TL) phenomenon, where light emission from a solid material is stimulated by the heating, which is preceded by excitation at low temperature. The excitation generates charge carriers. Most of them recombine immediately; however, some of them can escape the geminate recombination and remain in the localized sites traps. In the next step, the energy is released as visible light while the material is heating. The spectrally resolved thermoluminescence (SRTL) in the 15–300 K range is very useful for investigating the electroluminescence materials, especially with multicomponent systems, allowing the identification of radiative recombination centers [27].

**Figure 8:**
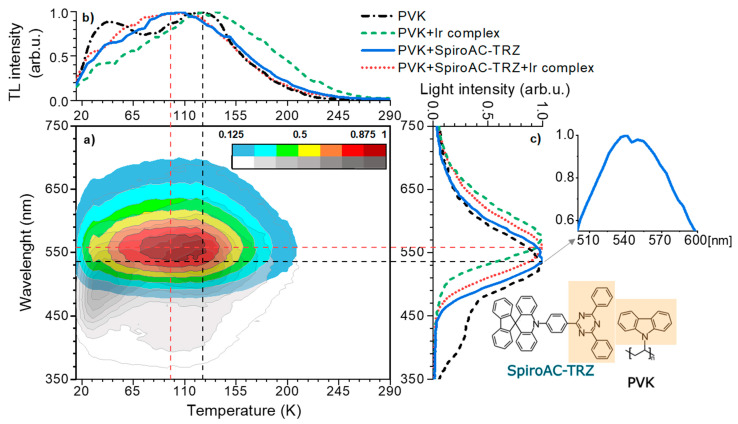
Spectrally resolved thermoluminescence (SRTL) spectra of layers of neat PVK, doped with 2 wt% of Ir, doped with 25 wt% of SpiroAC–TRZ and doped with 25 wt% SpiroAC–TRZ + 2 wt% Ir. (**a**) Thermoluminescence (TL) maps of layers of neat PVK and of PVK + 25 wt% SpiroAC–TRZ + 2 wt% Ir. (**b**) Normalized monochromatic TL curves recorded at emission maxima (λ~550 nm). (**c**) Isothermal spectra of emitted light recorded at temperature corresponding to TL maxima. Inset shows emission curve of PVK+25 wt% SpiroAC–TRZ layer, in range of 500–600 nm. Straight lines on SRTL map indicate: selected wavelength for monochromatic TL curves (horizontal lines) and selected temperature for isothermal spectra of emitted light (vertical lines).

In Figure 8, SRTL results obtained for three systems based on the PVK matrix are compared to the neat host layer. The monochromatic TL curves and the spectral distributions of the emitted light (at temperatures corresponding to TL maxima) are shown in Figure 8b,c, respectively. Two broad bands in the monochromatic TL spectrum can be distinguished for a neat PVK film. Both maxima have similar intensities, but the high-temperature peak is slightly higher than the one appearing at low temperature. This indicates that upon heating, the trapped charge carriers are subsequently released from two different trapping sites of similar densities. It was previously described in the literature that the high temperature maximum (around 125–130 K) originates from excimer traps and the second one, below 50 K, is connected with monomeric triplet states [28]. Moreover, the partial heating experiments proved that the two maxima correspond to the presence of two main trapping levels: around 50 and 200 meV [29]. Implementation of any dopant into PVK matrix causes quenching of the low-temperature maximum. This effect can be assigned to the lower population of trap sites related to the monomeric triplet states. It also reflects in isothermal spectra of emitted light at temperatures corresponding to TL maxima (Figure 8c). Accordingly, incorporation of any dopant into PVK results in disappearing of the short-wavelength emission, that is associated with the singlet excimers of carbazole groups (band with λ_max_~410 nm) and the monomeric triplet excitons (band with λ_max_~430 nm) (Figure 8c) [30]. Furthermore, PVK characterizes more intensive and broad emission, with the maximum at 530 nm, that is derived from the low energetic triplet excimers of fully overlapped carbazole groups [31]. Unfortunately, this broad band partly overlaps with the long-wavelength emission in the range of 450–750 nm, that is observed for the investigated three doped systems.

TL maps obtained for all investigated host–guest systems: PVK + 2 wt% Ir, PVK + 25 wt% SpiroAC–TRZ and PVK + 25 wt% SpiroAC–TRZ + 2 wt% Ir are similar. Thus, in Figure 8a TL maps are only depicted for layers of neat PVK and PVK + 25 wt% SpiroAC–TRZ + 2 wt% Ir, in order to keep the picture transparent. Comparing the TL maps and emission spectra shown in Figure 8a,c, it can be assumed that the dopants create new centers of radiative recombination in PVK based layers. At the same time, the monochromatic TL curves, shown in part b of Figure 8, are different for each system. As was mentioned above, a less intense TL signal at low temperatures indicates a smaller population of shallow traps for the doped PVK matrix. In addition to this, some changes in the position of high-temperature TL maximum as well as in the shape of curves can be noticed. Incorporation of Ir complex into PVK matrix (solid green curve in Figure 8b) results in the broadening of the main band of the monochromatic TL curve. The shape of this curve in the range of higher temperatures (up to 240 K) indicates a meaningful impact of deep traps created on the Ir complex molecules. Furthermore, luminescence of the layer with only Ir doped into the matrix is significantly red-shifted with the narrower full width at half maximum (see Figure 8c). Taking all these differences into account, it can be established that the emission from Ir is dominant. A similar effect was observed in the case of its incorporation into the PVK:PBD matrix [16].

Implementation of only TADF material into the PVK matrix results in a broad monochromatic TL spectrum with only one maximum at c.a. 100 K. A shift of the position of the main TL maximum from 125–130 K for neat PVK to 100 K for PVK with 25 wt% SpiroAC–TRZ indicates that in such a doped system, shallower traps dominate, contrary to those ones found in the pure matrix. These shallower traps maybe attributed to triplet exciplexes formed by carbazole groups and acceptor parts of SpiroAC–TRZ molecules, similarly to the case of the PVK:PBD blend [28]. This suggestion is confirmed by the emission spectrum recorded in the temperature related to the TL maximum. In isothermal luminescence spectrum (measured at 100 K) (blue curve in Figure 8c), one can distinguish two overlapping bands with maxima at ~535 nm and ~555 nm. Moreover, a slight increase of the half width of the total emission in comparison to the main emission band of the neat PVK matrix may also suggest the overlap of two emission bands. The first can be associated with low energetic excimeric triplet states of carbazole groups and the second one (~555 nm) with exciplex triplet states created between carbazole and triazine moieties. The formation of such intermolecular exciplexes between triazine derivative and carbazole groups of PVK has been previously observed [32,33]. The presence of them was manifested by emission band the maximum of which was observed at λ_max_ 520–530 nm in PL and EL spectra for layers of a blend of 2,4,6-Tris [3-(diphenylphosphinyl) phenyl]-1,3,5-triazine (POT2T) with PVK. The emission spectra of PVK + SpiroAC–TRZ are very similar to that recorded by Lin et al [17] for pure TADF film (λ_max_ 480 nm). However, a small red shift is observed when the doping concentration of TADF molecules increases. This may indirectly indicate the presence of some emissive states associated with intermolecular interaction in PVK:SpiroAC–TRZ film. Nonetheless, SRTL results coincide very well with the effect of EL spectrum widening in the long-wave range for PVK with a high content of SpiroAC–TRZ (Figure S1b) and may confirm the suggested probability of PVK:SpiroAC–TRZ exciplexes formation. Similar spectral evolution, explained by exciplex creation, was described for the platinum complex in TADF hosts containing the triazine unit [34].

The comparison of TL results of studied layers indicates that the presence of 25 wt% SpiroAC–TRZ in PVK affects the ability to occupy trap states on the Ir complex molecules by charge carriers. In addition, the maximum of isothermal emission for the ternary composition is situated between the maxima for the one doped systems: PVK + 25 wt% SpiroAC–TRZ and PVK + 2 wt% Ir (Figure 8c). Furthermore, the full width at half maximum of this band is comparable to the one of PVK with SpiroAC–TRZ. Accordingly, it can be assumed that both exciplex triplet states and excited states of Ir complex molecules play a significant role in total SRTL emission of the layer consisted of PVK:SpiroAC–TRZ and Ir.

**Figure 9:**
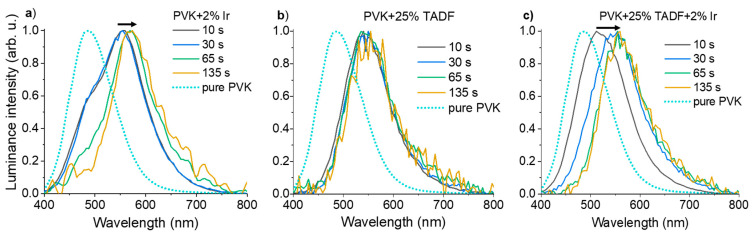
Spectra of isothermal luminescence decays registered in a long time scale at 20 K. Layers of: (**a**) PVK + 2 wt% Ir, (**b**) PVK + 25 wt% SpiroAC–TRZ, (**c**) PVK + 25 wt% SpiroAC–TRZ + 2 wt% Ir.

During the optical excitation at very low temperatures, some part of all generated geminate electron–hole pairs becomes captured by various kinds of traps due to significantly limited participation of phonons. Nevertheless, a substantial part of the generated charge pairs recombines radiatively with some delay. Therefore, when the excitation was switched off, isothermal luminescence at 20 K decayed within several minutes. In Figure 9, the isothermal luminescence spectra for investigated layers are presented in relation to the signal of pure PVK film. These emission spectra were recorded before the SRTL run, after excitation at 337 nm wavelength (similarly as for photoluminescence experiments done at room temperature) and for selected delay times after switching off the excitation source. This observed luminescence is the result of deactivation of long-lived excited states at low temperature, and thus, the presence of very shallow traps. In Figure 9a, spectra of PVK doped with 2 wt% of Ir, measured at 20K, are shown. Two peaks can be distinguished, the dominant one in the range of 500–700 nm (with a maximum at ~570 nm) and the second one with a maximum at about 485 nm. The short wavelength band contribution is significant just after the excitation is switched off and disappears with the time. This high energetic emission is similar to the isothermal spectrum of pure PVK layer recorded at 20 K, associated with both monomeric and excimeric triplet states (high and low energetic) of carbazole groups [35]. The prevailing band resembles the EL spectrum of OLED based on PVK + 2 wt% Ir emitting layer (Figure S5c) that was attributed to triplet excitons of the iridium complex. It is worth adding that the emission decay of the iridium doped PVK layer is faster than that observed for pure PVK. Thus, the triplet excitons of Ir complex play a dominant role in the radiative recombination.

As can be seen in Figure 9b, the position and the shape of the emission band do not change with the time for the system of PVK + 25 wt% SpiroAC–TRZ. Luminescence measured at 20 K occurs in the same wavelength range as the isothermal luminescence recorded at 105 K. The lack of emission related to PVK excited states confirms that implementation of TADF inhibited the creation of PVK excimers. The observed emission can be mostly attributed to exciplexes formed between triazine and carbazole groups. The more complicated situation is observed for the three-component system of PVK + 25 wt% SpiroAC–TRZ + 2 wt% Ir (see Figure 9c). Initial emission (after 10 s) can be associated with monomeric and excimeric PVK triplet states, as well as PVK:SpiroAC–TRZ exciplexes. The contribution of PVK excited states, to the emission recorded over a longer time scale, is getting smaller. Finally, isothermal luminescence measured after more than 1 min is connected with the emission of PVK:SpiroAC–TRZ exciplexes with significant contribution of iridium complex triplet states.

### 3.3. Exciton Energy Transfer

**Figure 10:**
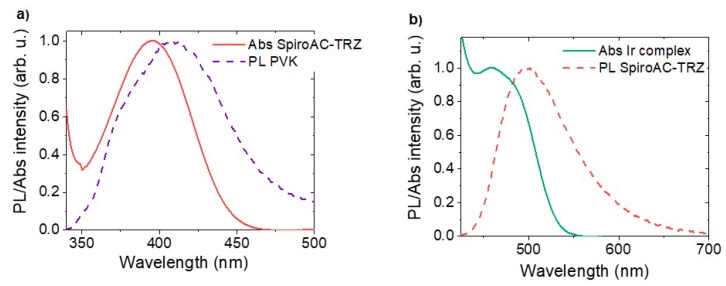
(**a**) Normalised PL spectrum of neat PVK matrix (film) and absorption spectrum of TADF emitters (chlorobenzene solution) (**b**) Normalised PL spectra of TADF emitter (embedded in PVK matrix) with absorption spectrum of Ir complex (chlorobenzene solution).

Generally, excitons formed on host molecules can be transferred to the guest via electron exchange mechanism proposed by Dexter [36] or, which is more probable in the system with small dopant concentration, through Förster resonance energy transfer (FRET). The second mechanism is long-range (even up to 10 nm) dipole–dipole coupling of the host and the guest molecules [37,38]. In the studied systems, both mechanisms are probably involved in energy exchange but Forster transfer dominates because of its long-range nature. However, the Dexter mechanism cannot be neglected in the case of energy exchange between PVK and SpiroAC–TRZ molecules. In both cases, spectral overlap between host emission and guest absorption (see Figure 10) favors efficient energy transfer [39].

To analyze the exciton energy transfer efficiency in the multicomponent emissive system of PVK + 25 wt% SpiroAC–TRZ + 2 wt% Ir, first, the simplified structure without the Ir complex (PVK with SpiroAC–TRZ) was considered (Figure 10a). The calculations of Förster energy transfer parameters presented in Table 2 were performed according to the following Equations (1)–(3) [40]

(1) $$R_{F}=0.0211\cdot \sqrt[{6}]{\frac{k^{2}}{n^{4}}\cdot QY_{D}\cdot J_{o}}\;\left[\mathrm{nm}\right],$$

where *R*_F_—the characteristic Förster radius, *k*^2^—the orientation factor, *n*—the refractive index of the medium in the wavelength range where spectral overlap is significant, *QY*_D_—the quantum yield of donor photoluminescence, and *J*_o_—the degree of spectral overlap between donor fluorescence spectrum and acceptor absorption spectrum, given below in terms of wavelength λ [40]:

(2) $$J_{o}=\overset{+\infty }{\underset{0}{\int }}f_{D}\left(\lambda \right)\varepsilon_{A}\left(\lambda \right)\lambda^{4}d\lambda ,$$

where *f*_D_(λ)—the donor fluorescence spectrum normalized, so that the integral is equal to unity, and *ε*_A_(λ)—the scaled acceptor absorption spectrum to its maximum of molar extinction coefficient.

The efficiency of Förster energy transfer from the donor to the acceptor molecules is estimated as follows: [41,42]

(3) ηF=11+(RDARF)6

where $R_{\mathrm{DA}}$—the average separation distance between the donor and the acceptor molecules, estimated pursuant to previously published procedure [30].

The efficiency of Förster energy transfer from PVK to SpiroAC–TRZ was estimated as 88% (Table 2), whereas PL measurement reveals complete transfer (emission in Figure S1 can be assigned only to TADF). This discrepancy is connected with the contribution of Dexter energy transfer due to high SpiroAC–TRZ concentration. The energy transfer by both mechanisms, and possibly contributing exciton diffusion process, explain the lack of emission band from the PVK matrix in PL spectra of PVK:SpiroAC–TRZ thin layers. Diversely, in a case of energy transfer from SpiroAC–TRZ to the phosphorescent emitter, FRET will dominate due to small Ir complex concentration. To analyze this Förster mechanism, the donor ability of SpiroAC–TRZ was first investigated in PVK surroundings. Therefore, the emission spectrum of SpiroAC–TRZ for estimation of spectral overlap integral (*J*_o_) (Figure 10b) as well as the quantum yield of donor photoluminescence (*QY*_D_) were determined for the layer of PVK + 25 wt% SpiroAC–TRZ (Table 2).

In the case of exciton energy transfer from SpiroAC–TRZ to Ir complex, the theoretical efficiency of the transfer by Förster mechanism is only 21%, whereas the emission from Ir complex prevails in PL spectrum (Figure 4a). Proper estimation of the energy transfer in the real system is impeded due to the overlap of TADF and Ir complex emission bands. In fact, this process is much more effective than the theoretical calculations predict. The reason of that may be mentioned Dexter energy transfer between PVK and SpiroAC–TRZ as well as the presence of carbazole group: triazine unit of TADF molecule exciplexes. The triplet and singlet excitons of these individuals (S_exc_, T_exc_) can also participate in the energy transfer mechanism. Moreover, the energy transfer is enhanced by exciton diffusion processes, that reduces the actual average distance between donor and acceptor molecules (R_DA_) [35]. The explanation of it is similar to that proposed for the systems of PVK:PBD matrix doped by small concentration of an Ir complex. However in this case the triplet excitons of PBD and PVK:PBD exciplexes do not take part in energy transfer to iridium complex molecules, they are lost because of large singlet–triplet splitting energy and low probability of RISC [28].

### 3.4. Comparison of PL, EL and SRTL Results

**Figure 11:**
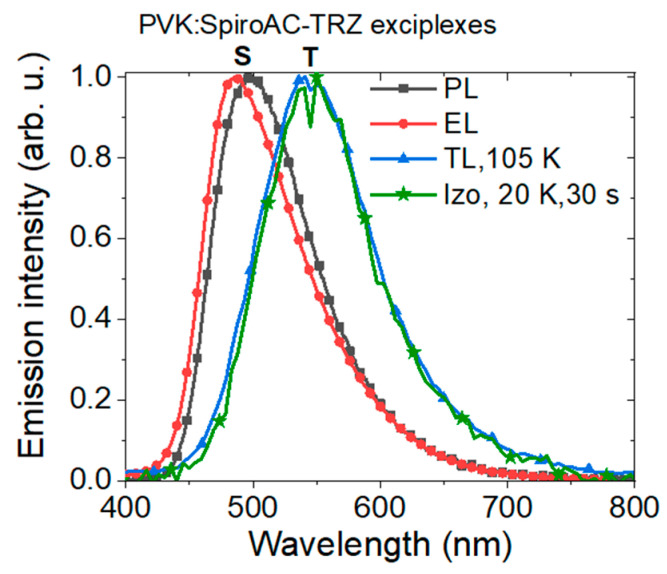
Spectra of PVK + 25 wt% SpiroAC–TRZ recorded during PL, EL and TL experiments.

The comparison of PL, EL and TL spectra of the PVK + 25 wt% SpiroAC–TRZ system (Figure 11) shows a variety of recombination states, that take part in radiative processes in these phenomena. In PL and EL spectra, the emission (λ_max_~490 nm) from the excited states of TADF molecules [17] is prevailing and long wavelength emission is observed only as band tail; whereas in SRTL spectra, the luminescence of lower energy (with λ_max_~550 nm), assigned to triplet states of carbazole:triazine exciplex, definitely dominates. A similar scenario with the contribution of different excited states in EL and TL phenomena was stated before for the PVK:PBD system [27]. In the case of PVK:PBD blend, the singlet exciplexes that operate in the EL phenomenon reveal emission peak at 425–430 nm, while the radiative deactivation of triplet exciplexes with the emission maximum at 550 nm is observed in TL.

**Figure 12:**
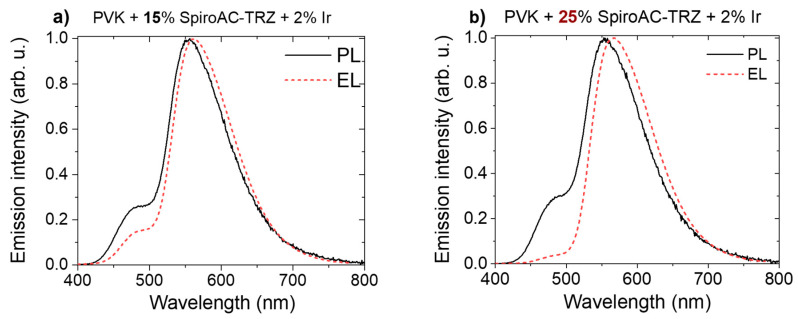
EL spectra (recorded at 1 mA/cm^2^) and PL spectra of PVK + 15 or 25 wt% SpiroAC–TRZ + 2 wt% Ir.

The temperature is a critical factor for the radiative recombination mechanisms observed in EL and TL phenomena. During the optical excitation at low temperature (TL experiment), part of the generated electron–hole pairs is trapped on various kinds of species but mostly on formed exciplexes. Only when thermal energy is delivered to the sample are the charge carriers released from the traps and next, can directly recombine radiatively by the intersystem crossing or created excitons can be transferred on other species (molecules). On the contrary, in the electroluminescence phenomenon, at room temperature, the singlet states act as radiative recombination centers, whereas triplet exciplexes preferably operate as transport and/or trapping centers with an extremely short time of charge carriers stay. Such a mechanism could be proposed for PVK:PBD. However, when PVK is mixed with TADF SpiroAC–TRZ, molecules with small S-T energy splitting and high RISC probability, the monomer as well as exciplex excitons can be involved in electroluminescence phenomena. Moreover, in the case of ternary systems, formed triplet exciplexes of PVK:SpiroAC–TRZ promote long-range transfer of exciton energy to the emitter and simultaneously, can improve the transport process of charge carriers. This statement is confirmed by the results presented in Figure 12. In the EL spectrum for a system with a higher SpiroAC–TRZ content (25 wt%), the contribution of the TADF emission is negligible, although in PL it is comparable with the one recorded for the layer of PVK + 15 wt% SpiroAC–TRZ. This divergence can be related to the presence of a larger exciplexes population under the electric field which consequently can lead to more efficient energy transfer to the Ir complex molecules. These observations may suggest the electroplex formation, more probable in a layer with 25% of TADF. The term electroplex was introduced to describe an excited state complex which can be achieved only under an electric field and is not observed in PL [43]. The presence of such species can effectively support the excitation energy transfer and might be the reason for differences in photo- and electroluminescence. That would also explain EL spectrum broadening with increasing doping concentration of TADF molecules in the PVK matrix (Figure S1b) as well as the small blue shift of EL maximum observed for the PVK + 25 wt% Spiro-AC-TRZ + 2 wt% Ir complex in comparison to the layers without TADF assistance (Figure S5c).

In view of the above, it seemed reasonable to compare the results obtained for OLEDs with the PVK:PBD matrix used instead of pure PVK. Generally, the introduction of PBD or TADF molecules to the PVK matrix enhances not only the efficiency but also the operational stability of diodes with phosphorescent Ir (III) complex (Table S2 and Figure S5). The normalized luminance of OLEDs as a function of operation time at a constant current show that addition of PBD or TADF extends the operational lifetime of OLEDs (defined as operation time at which the luminance drops to 50% of the initial value) two or five times, respectively. The improvement caused by TADF assistance in PVK is significantly greater than that obtained in the case of PVK:PBD matrix doped by 2 wt% Ir complex (Figure S5a,b and [16]), in which PBD incorporation resulted mainly in charge carriers balance improvement. Very similar working parameters, i.e., *L*_max_ = 14,000 cd/m^2^ and *η*_max_ = 18 cd/A, were obtained for OLEDs with emissive layer PVK+TADF + Ir complex or PVK:PBD +TADF + Ir complex (Table S2). However, in the latter case, the improvement of device performance caused by TADF assistance is not such spectacular (efficiency increased from 13 to 18 cd/A) (Table S2). Moreover, OLEDs with PVK + SpiroAC–TRZ + Ir as an emissive layer have a lower switching voltage compared to the OLEDs based on PVK:PBD or PVK. This results from easier injection of electrons directly to the LUMO level of SpiroAC–TRZ and more effective creation of exciplexes, the energy of which is lower than the energy of PVK:PBD exciplexes and S_1_ state of PVK.

Similar results obtained for the incorporation of TADF assistant into PVK or PVK:PBD matrix might lead to the conclusion that SpiroAC–TRZ used to improve the triplet harvesting can additionally play a similar role as PBD molecules in the system of PVK:PBD, i.e., act as an electron transporting material and create exciplexes [28]. The above discussion concerning the increase in the efficiency and stability of produced OLEDs is in line with the mechanism called “exciplex-triplet energy transfer” (ExTET) introduced by the Yamazaki group [44,45]. They discussed the effect of exciplex formation on quantum efficiency and drive voltage in the ExTET OLED by combining various exciplex species with a phosphorescent and also fluorescent emitter. It was shown the exciplexes can be used as a medium of energy transfer when the energy of their triplet state is close to T_1_ of the guest. Utilizing this energy transfer from exciplex to emitter allows them to achieve high-performance phosphorescent OLED.

In view of all of the above, it seems that in the system of “matrix → TADF → phosphorescent emitter” (PVK + 25 wt% SpiroAC–TRZ + 2 wt% Ir), the dominant mechanism responsible for the efficient emission from Ir complex is not the process of charge carriers trapping on the emitter but the transfer of exciton energy. That is in contrast to previously obtained results for host–guest systems consisting only from polymer matrix and Ir complex [16,46].

## 4. Conclusions

In summary, we demonstrated that owing to the TADF assistance, a significant improvement of efficiency and operational stability of devices can be achieved in solution-processed OLEDs with the following structure: ITO/PEDOT:PSS (20 nm)/LEL (65 nm)/Bphen (40 nm)/Liq (2 nm)/Ag (100 nm), where the emissive layers were composed of: PVK + 2 wt% Ir or PVK + 25 wt% SpiroAC–TRZ + 2 wt% Ir. Introducing SpiroAC–TRZ as assistant dopant resulted in the lowering of turn-on voltage (to 6 V), rising brightness (up to 18,000 cd/m^2^), stabilizing flowing current and four–fold increase of the current efficiency (up to 23 cd/A) with respect to the corresponding device without TADF assistance. Furthermore, EL completely occurs from Ir complex, which confirms appropriate materials adjustment which ensures the effective excitation energy transfer to the phosphorescent emitter. The analysis of photo-, electro- and thermoluminescence results revealed that PVK:SpiroAC– TRZ, exciplexes/electroplaxes, similarly to PVK:PBD, play an important role in this process. This enhances energy transfer to target emitter and additionally improves the transport of charge carriers in system, finally providing enhancement of OLEDs performance in comparison to their conventional phosphorescence-based counterparts.

## Figures and Tables

**Table 1:** Photophysical parameters of emissive layers of PVK with different SpiroAC–TRZ (thermally activated delayed fluorescence (TADF) assistant dopant) and Ir concentrations as well as organic light emitting diodes (OLED) parameters based on such systems. OLED configuration: ITO/PEDOT:PSS (20 nm)/light emitting layer (LEL) (70 nm)/TPBi (20 nm)/Liq (2 nm)/Ag (100 nm).

Emissive System	λ_PLfilm_ (nm)	QY (%)	λ_EL_ (nm)	L_max_ (cd/m^2^)	η_max_ (cd/A)
PVK + 15 wt% SpiroAC–TRZ	486	46	484	3500	4.3
PVK + 20 wt% SpiroAC–TRZ	487	46	488	5100	5.4
PVK + 25 wt% SpiroAC–TRZ	490	46	486	8100	7.2
PVK + 30 wt% SpiroAC–TRZ	494	42	488	6300	5.3
PVK + 15 wt% SpiroAC–TRZ + 1 wt% Ir	494, 548	26	486, 555	8000	11
PVK + 15 wt% SpiroAC–TRZ + 2 wt% Ir	493, 556	27	487, 562	11,000	16
PVK + 15 wt% SpiroAC–TRZ + 3 wt% Ir	492, 556	21	488, 565	9000	13
PVK + 25 wt% SpiroAC–TRZ + 2 wt% Ir	491, 553	20	487, 567	14,000	18

**Table 2:** Förster energy transfer parameters of active layers with SpiroAC–TRZ as the guest and as the assistant dopant with target phosphorescent emitter.

Emissive Layer	*QY*_D_ (%)	*n* * (−)	*ε_A_* (10^3^ M^−1^·cm^−1^)	*J*_o_ (10^13^ nm^4^·M^−1^·cm^−1^)	*R*_F_ (nm)	*R*_DA_ (nm)	*η*_F_ (%)
PVK + 25wt% SpiroAC–TRZ	11	1.822	2.8 ^#^	3.59	1.66	1.19	88
PVK + 25wt% SpiroAC–TRZ + 2 wt% Ir	46	1.729	2.7	5.82	2.37	2.95	21

* The refractive index of the emissive layer estimated from ellipsometry measurements; ^#^ taken from the reference [17].

## Data Availability

Not applicable.

## Supplementary Materials

The following are available online at https://www.mdpi.com/article/10.3390/polym13071148/s1, Figure S1: (a) PL spectra of PVK layers with different SpiroAC – TRZ concentration (10–30 wt%); (b) EL spectra of the investigated systems. Figure S2: Luminance–voltage characteristics of OLEDs based on PVK doped with different SpiroAC–TRZ (TADF) concentration: OLED configuration: ITO/PEDOT:PSS (20 nm)/LEL (70 nm)/TPBi (20 nm)/Liq (2 nm)/Ag (100 nm), Figure S3: Current efficiency dependencies on current density of OLEDs with the following structure: ITO/PEDOT:PSS (20 nm)/PVK + 25 wt% SpiroAC–TRZ + 2 wt% Ir (75 nm)/Liq (2 nm)/Ag (100 nm). (a) 20 nm TPBi, (b) 40 nm TPBi; Figure S4: Work parameters of OLEDs with the structure: ITO/PEDOT:PSS/PVK + 25 wt% SpiroAC–TRZ + 2 wt% Ir (65 nm)/TPBi or Bphen (40 nm)/Liq (2 nm)/Ag (100 nm). (a) Current density–voltage–luminance (*J*–*V*–*L*) characteristics; (b) Current efficiency dependencies on current density; Figure S5: Work parameters of OLEDs based on following emissive layers: PVK + 2 wt% Ir complex, PVK+PBD + 2 wt% Ir complex and PVK + 25 wt% SpiroAC–TRZ + 2 wt% Ir complex. Device structure: ITO/PEDOT:PSS (20 nm)/LEL (65 nm)/Bphen (40 nm)/Liq (2 nm)/Ag (100 nm). (a) Current density–voltage–luminance (*J*–*V*–*L*) characteristics; Current density–voltage characteristics (left) and luminance–voltage characteristics (right); (b) Current efficiency dependencies on current density. (c) EL spectra, (d) Normalized EL decay curves of the investigated OLEDs as a function of operational time at a preliminary luminance (*L*_0_ = 1000 cd/m^2^); measured in the constant current regime; Table S1: Photophysical parameters of emissive layers of PVK with different SpiroAC–TRZ and Ir concentrations as well as OLED parameters based on such systems; Table S2: Operation parameters of OLEDs based on different emissive systems. OLED configuration: ITO/PEDOT:PSS (20 nm)/LEL (65 nm)/TPBi (40 nm)/Liq (2 nm)/Ag (100 nm).
