# Supplementary material for: Effect of TADF Assistance on Performance Enhancement in Solution Processed Green Phosphorescent OLEDs
Source: Polymers (Basel). 2021 Apr 2;13(7):1148. doi: 10.3390/polym13071148 (PMC8038252; doi:10.3390/polym13071148)
Supplement: Supplementary file 1 [file polymers-13-01148-s001.pdf]

polymers

MDPI

Supplementary Material

Effect of TADF Assistance on Performance Enhancement in Solution Processed Green Phosphorescent OLEDs

Ewelina Witkowska [1,2], Gabriela Wiosna-Salyga [1,*], Ireneusz Glowacki [1], Tung-Huei Ke [2,*], Pawel Malinowski [2] and Paul Heremans [2]

[1] Department of Molecular Physics, Lodz University of Technology, Zeromskiego 116, 90-924 Lodz, Poland; ewelina.witkowska@p.lodz.pl (E.W.); ireneusz.glowacki@p.lodz.pl (I.G.)

[2] IMEC, Kapeldreef 75, B-3001 Leuven, Belgium; pawel.malinowski@imec.be (P.M.); paul.heremans@imec.be (P.H.)

* Correspondence: gabriela.wiosna-salyga@p.lodz.pl (G.W.-S.); Tung.Huei.Ke@imec.be (T.-H.K.)

Supporting results

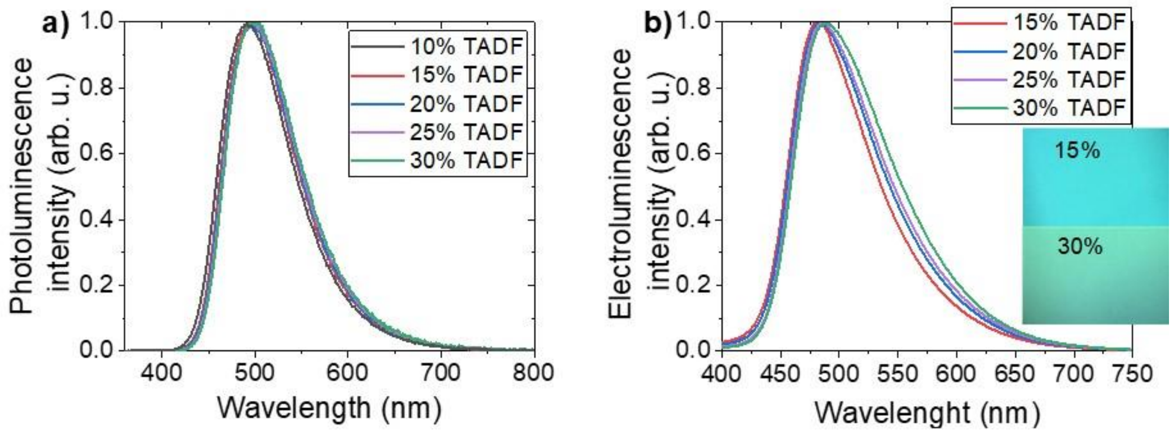

Figure S1. a) PL spectra of PVK layers with different SpiroAC-TRZ concentration (10–30 wt%); **b**) EL spectra of the investigated systems. Pictures of working devices with emissive layers of PVK+ 15 and 30 wt% SpiroAC–TRZ are the insets.

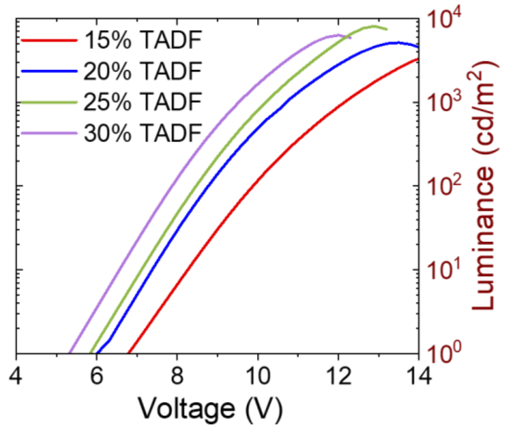

Figure S2. Luminance–voltage characteristics of OLEDs based on PVK doped with different SpiroAC–TRZ (TADF) concentration: OLED configuration: ITO/PEDOT:PSS (20 nm)/LEL (70 nm)/TPBi (20 nm)/Liq (2 nm)/Ag (100 nm).

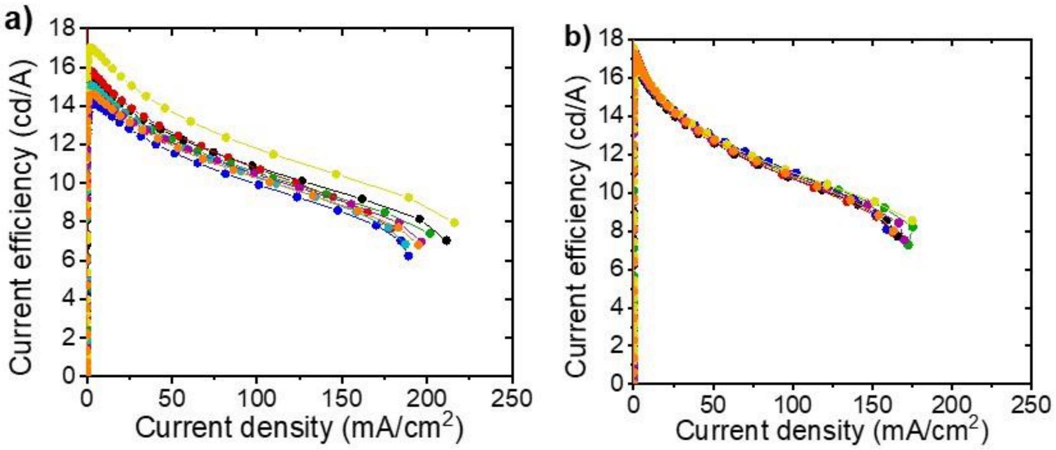

Figure S3. Current efficiency dependencies on current density of OLEDs with the following structure: ITO/PEDOT:PSS (20 nm)/PVK+25 wt% SpiroAC–TRZ+2 wt% Ir (75 nm)/ Liq (2 nm)/ Ag (100 nm). a) 20 nm TPBi, b) 40 nm TPBi.

Table S1. Photophysical parameters of emissive layers of PVK with different SpiroAC–TRZ and Ir concentrations as well as OLED parameters based on such systems.

Emissive System	λ_{PLfilm} [nm]	QY [%]	λ_{EL} [nm]	L_{max} [cd/m^2]	η_{max} [cd/A]
OLED configuration: ITO/PEDOT:PSS (20 nm)/LEL (70 nm)/ TPBi (20 nm)/Liq (2 nm)/Ag (100 nm)					
PVK + 15 wt% SpiroAC–TRZ	486	46	484	3500	4.3
PVK + 20 wt% SpiroAC–TRZ	487	46	488	5100	5.4
PVK + 25 wt% SpiroAC–TRZ	490	46	486	8100	7.2
PVK + 30 wt% SpiroAC–TRZ	494	42	488	6300	5.3
PVK + 15 wt% SpiroAC–TRZ + 1 wt% Ir	494, 548	26	486, 555	8000	11
PVK + 15 wt% SpiroAC–TRZ + 2 wt% Ir	493, 556	27	487, 562	11,000	16
PVK + 15 wt% SpiroAC–TRZ + 3 wt% Ir	492, 556	21	488, 565	9000	13
PVK + 25 wt% SpiroAC–TRZ + 1 wt% Ir	490, 545	26	-	-	-
PVK + 25 wt% SpiroAC–TRZ + 2 wt% Ir	490, 553	20	487, 565	14,000	16
PVK + 25 wt% SpiroAC–TRZ + 3 wt% Ir	490, 561	19	-	-	-
OLED configuration: ITO/PEDOT:PSS (20 nm)/LEL (70 nm)/TPBi (40 nm)/ Liq (2 nm)/Ag (100 nm)					
PVK+1 wt% Ir	555	14	566	6000	10.7
PVK + 25 wt% SpiroAC–TRZ	490	46	488	7000	9.5
PVK + 25 wt% SpiroAC–TRZ + 2 wt% Ir	490, 553	20	567	14,000	18
OLED configuration: ITO/PEDOT:PSS (20 nm)/LEL (65 nm)/Bphen (40 nm)/ Liq (2 nm)/Ag (100 nm)					
PVK+1 wt% Ir	555	14	576	870	6.9
PVK + 25 wt% SpiroAC–TRZ + 2 wt% Ir	490, 553	20	565	18,500	23.8

Table S2. Operation parameters of OLEDs based on different emissive systems. OLED configuration: ITO/PEDOT:PSS(20 nm)/LEL(65 nm)/TPBi(40 nm)/Liq(2 nm)/Ag(100 nm).

	λ_{EL} [nm]	L_{max} [cd/m^2]	η_{max} [cd/A]	*EQE* [%]
PVK + 2 wt% Ir complex	566	6000	10.7	3.7
PVK + 25 wt% SpiroAC–TRZ	488	7000	9.5	4.0
PVK + 25 wt% SpiroAC–TRZ + 2 wt% Ir complex	566	14,000	18.0	6.2
PVK:PBD + 2 wt% Ir complex	568	12,000	13.0	4.5
PVK:PBD + 30 wt% SpiroAC–TRZ	496	7,000	9.5	3.7
PVK:PBD + 30 wt% SpiroAC–TRZ + 2 wt% Ir complex	562	14,000	18.0	6.1

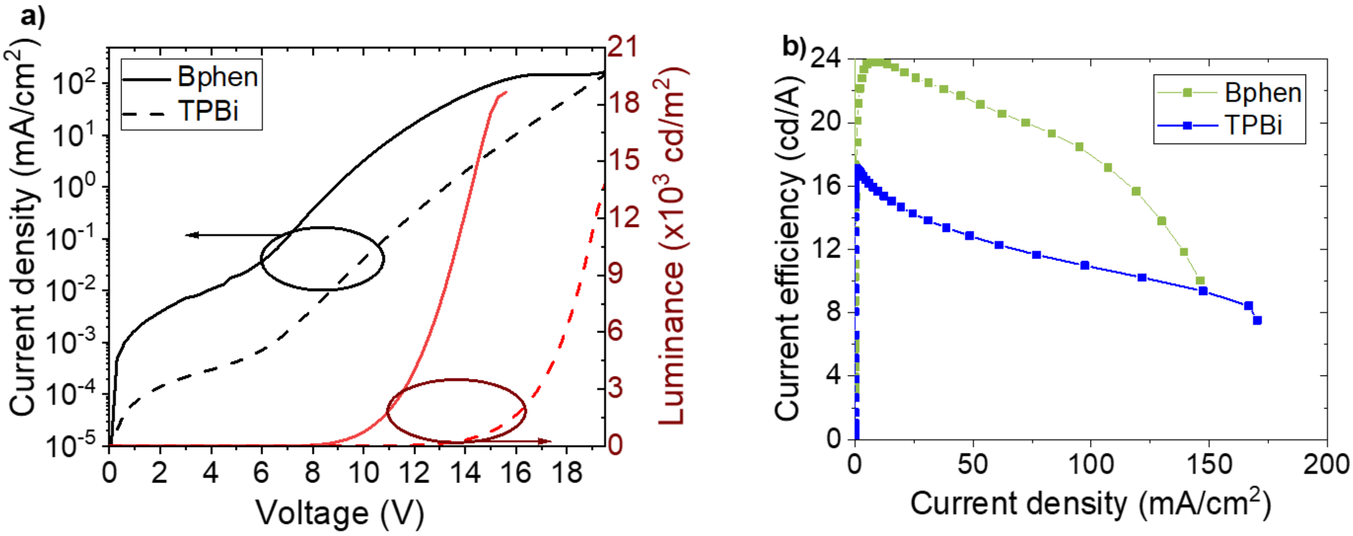

Figure S4. Work parameters of OLEDs with the structure: ITO/PEDOT:PSS /PVK+25 wt% SpiroAC–TRZ+2 wt% Ir (65 nm) /TPBi or Bphen(40 nm)/ /Liq(2 nm)/ Ag(100 nm).**a**) Current density–voltage–luminance (J–V–L) characteristics; **b**) Current efficiency dependencies on current density.

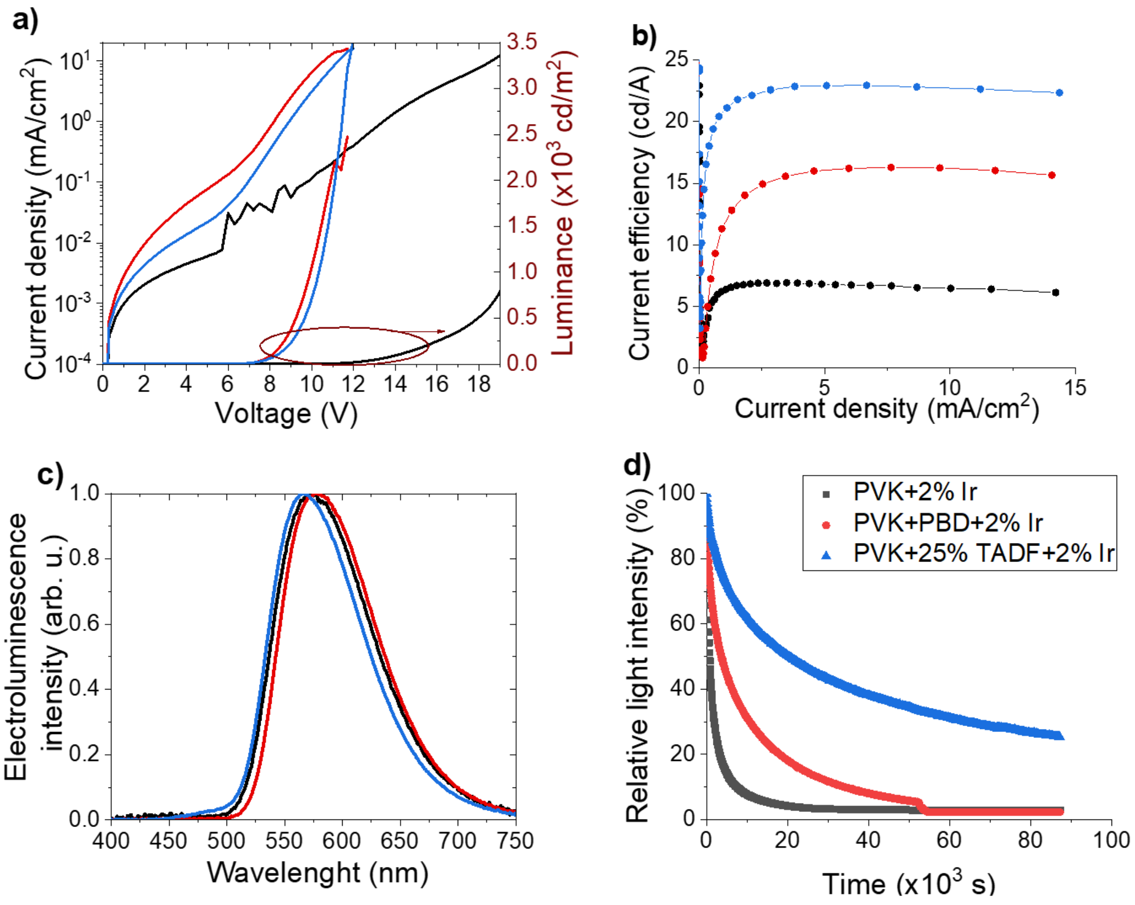

Figure S5. Work parameters of OLEDs based on following emissive layers: PVK + 2 wt% Ir complex, PVK+PBD + 2 wt% Ir complex and PVK + 25 wt% SpiroAC–TRZ +2 wt% Ir complex. Device structure: ITO/PEDOT:PSS(20 nm)/LEL(65 nm)/Bphen(40 nm)/Liq(2 nm) /Ag(100 nm). a) Current density–voltage–luminance (*J*–*V*–*L*) characteristics; Current density–voltage characteristics (left) and luminance–voltage characteristics (right); b) Current efficiency dependencies on current density. c) EL spectra, d) Normalized EL decay curves of the investigated OLEDs as a function of operational time at a preliminary luminance (L_0 = 1000 cd/m^2); measured in the constant current regime.
